# An IBeacon-Based Location System for Smart Home Control

**DOI:** 10.3390/s18061897

**Published:** 2018-06-11

**Authors:** Qinghe Liu, Xinshuang Yang, Lizhen Deng

**Affiliations:** 1 Bell Honors School, Nanjing University of Posts and Telecommunications, Nanjing 210023, China; liuqhxueshu@gmail.com (Q.L.); agnes.eyre@gmail.com (X.Y.); 2 School of communication and information engineering, Nanjing University of Posts and Telecommunications, Nanjing 210023, China

**Keywords:** intelligent control, indoor location, fingerprint matching, iBeacon, PDR

## Abstract

Indoor location and intelligent control system can bring convenience to people’s daily life. In this paper, an indoor control system is designed to achieve equipment remote control by using low-energy Bluetooth (BLE) beacon and Internet of Things (IoT) technology. The proposed system consists of five parts: web server, home gateway, smart terminal, smartphone app and BLE beacons. In the web server, fingerprint matching based on RSSI stochastic characteristic and posture recognition model based on geomagnetic sensing are used to establish a more efficient equipment control system, combined with Pedestrian Dead Reckoning (PDR) technology to improve the accuracy of location. A personalized menu of remote “one-click” control is finally offered to users in a smartphone app. This smart home control system has been implemented by hardware, and precision and stability tests have been conducted, which proved the practicability and good user experience of this solution.

## 1. Introduction

With the rapid development of Wireless Sensor Networks (WSNs) and IoT technologies [1,2], smart home systems are also advancing. People are coming to realize the importance of time and pay attention to more efficient lives. The smart home concept was born out of such a trend to bring us more convenience, and they are popular and well received [3]. Smart networks and mobile devices can bring great convenience for people’s daily life, and there are many methods of controlling electrical devices. The most traditional method is to set appliances manually, but it is also the most inefficient method. Some studies have proposed to control the appliances by searching menus. However, as more and more appliances are being joined in our home, this method of common-menu control is likely to lead to an unsatisfactory experience. It is put forward that a more efficient solution is to set a dynamic control menu [4]. Of course, while developing personalized menus, focusing on improve the ability of endurance and the accuracy of location will greatly optimize users’ experience.

Nowadays, though the most commonly used outdoor positioning technology is the GPS technology, it is not a reliable solution for indoor location, because satellite signals will be weakened severely when they go across buildings. Therefore, a variety of indoor location technologies have been proposed [1,5,6], for instance, ultrasonic, infrared ray, Radio Frequency Identification (RFID), Wi-Fi and so on. Among them, the RFID location technique can achieve high accuracy, but the cost is also high. Besides, these technologies share common shortcomings such as high energy consumption and limited endurance. For this reason, BLE based on the Bluetooth 4.0 standard came into being [7,8], with a longer battery life and extraordinary anti-attenuation characteristics. While pursuing the better signal technology, research on positioning algorithms is also advancing [9]. Currently, the received signal strength indicator (RSSI), carrier signal time of arrival (TOA), and time difference of arrival (TDOA) of the received signal are three commonly used standards for indoor location algorithms. In 2007, Stela proposed that the specific RSSI patterns at each location represented the location fingerprint of the particular location [10]. Location models have been applied in many fields in recent years. For example, a positioning algorithm was used in a fire emergency command system [11]. A smart home system using the location method of discretized RSSI-binarization matching was designed by Xiong [9]. However, the location accuracy of that algorithm needs to be improved. Kanzaki built a simulation system of optimal RSSI fingerprint matching [12], but did not adequately deal with the time-varying nature of RSSI. The accuracy still had room for improvement. Not long ago, a machine learning-based model was proposed that automatically updates the database [13], solving part of the time-varying problems to a certain extent, although the cost of that system is high and the update rate is limited.

Magnetometers and inertial sensors can detect three dimensional direction angles: deflection angle α, pitch angle β and roll angle γ [14,15]. In a definite space rectangular coordinate system, the deflection angle α represents the angle between the long side of the mobile phone and xOz plane, the pitch angle β represents the angle between the long side of the mobile phone and xOy plane, and the roll angle γ represents the angle between the short side of the mobile phone and xOy plane. Dong proposed a method based on hexahedron measurement to correct the error of magnetometers [16].

This paper puts forward the concept of fingerprint matching based on RSSI stochastic characteristics and the posture recognition model based on geomagnetic sensing, combined with Pedestrian Dead Reckoning (PDR) technology to solve the problems of time variation better and to improve the location accuracy. The system has been divided into five main parts: IBeacons, intelligent terminals, home gateway, Android App and web server. In this system, the core is the web server, which is a hub for connecting the various types of terminal equipment and beacons, executing the functions of location, storing dates, matching and updating the database. IBeacons mainly uses BLE4.0 low-energy technology to obtain a positioning coordinate system. On the Android App, we develop personalized menus for users, which can be used for remote pointing control of various household appliances. They are designed to simplify people’s lives and improve the user experience. Meanwhile, we propose a learning indoor location algorithm combining geomagnetic sensors and RSSI fingerprint dynamic matching. PDR technology is also used to correct errors to guarantee the accuracy of the location system.

## 2. System Design

**Figure 1:**
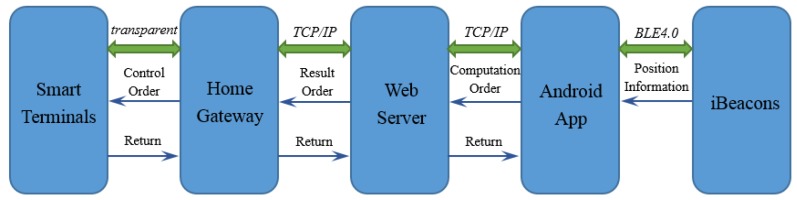
System Method Diagram.

In this section, the structure of the system and how the system works will be illustrated. This system connects an Android App to terminal devices via the web server and home gateway. Moreover, with the help of RSSI fingerprint matching and the PDR algorithm, users can choose and conveniently and precisely control the devices using a mobile phone. The following is the working principle of this system: as is shown in Figure 1, the proposed system has five main parts: smart terminals, home gateway, web server, Android app and iBeacons. The implementation of the system can be divided into the following six steps:

(1) IBeacons are installed at some fixed positions to establish a coordinate system. They broadcast signals to the Android app.
(2) The app on the mobile phone receives the signals, transmits the RSSI values of iBeacons and its own attitude data to the web server, and gives related control instructions. The “attitude data” means values of angles that can indicate the direction where the mobile phone is pointing.
(3) The web server analyses these data and instructions, estimates the phone’s location and attitude, and determines which device is being chosen.
(4) The web server sends data and instructions to the home gateway. These data and instructions contain the information of the electric devices that need to be controlled and the information of the specific control instructions.
(5) The home gateway analyzes the instructions, confirms the device number and sends instructions to the specific terminal device.
(6) At last, the chosen smart terminal operates under the control instructions.

It is notable that the smart terminal is a flexible access point of the IoT. Any electronic device only needs to install a universal terminal control module to connect to the network, which also brings great convenience to the promotion of IoT. The following sections will explain the design ideas and related device instructions for each of the five modules.

## 3. Implementation of System

### 3.1. Smart Terminals

**Figure 2:**
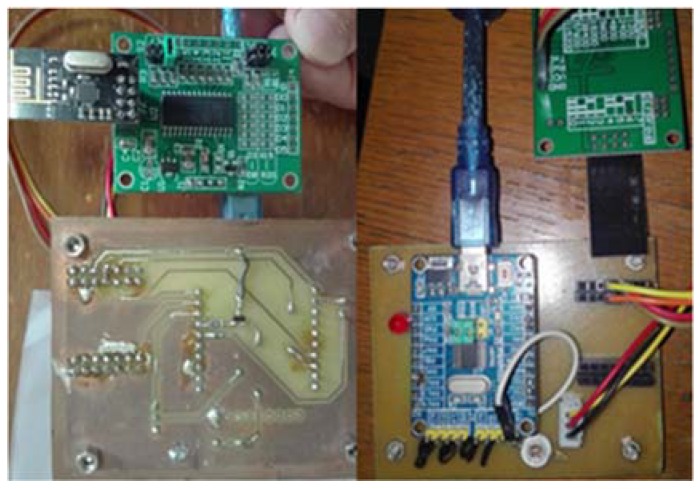
Smart Terminal.

Smart terminals are devices installed on the electric devices having four main functions, and the hardware of smart terminal is shown in Figure 2.

*Location information*: a RSSI receiving device was installed on the terminal, thus transmitting data to the web server via the home gateway. When the PDR device detects motion of electric devices, the web server will automatically use the location algorithm to determine the current device location. Therefore, it ensures that even if the location of devices has changed, the location information would automatically be updated and efficiency would be improved.

*Pointing indication*: When a user’s mobile device is pointing at an electric device, all control instructions of the mobile device are acting on the electric device. The device is at a being selected state and the LED will blink in order to tell the user that the device is being selected.

*Transparent communication*: Transparent communication is used to communicate to the home gateway, which is also used in the home gateway. This module uses several transparent modules for wireless communication in the local area network to transmit controlling and return data.

*Relay group*: A single relay is essentially a binary switch. The control signal is output via the high or low level of pins of the microcontroller. Several relays are combined into a multi-bit binary encoding group for multiple intelligent operations.

### 3.2. Home Gateway

**Figure 3:**
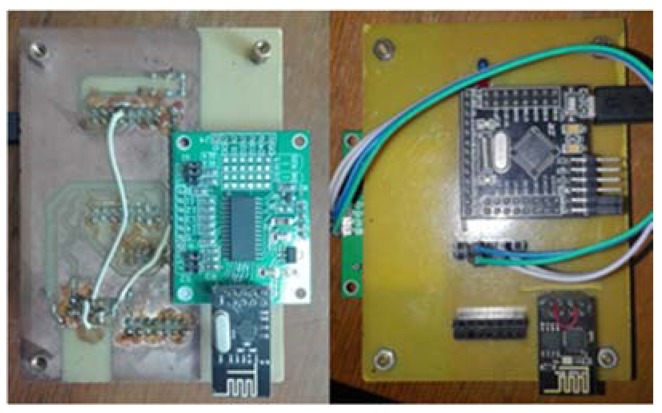
Home Gateway.

The home gateway has the same ability of transparent communication as the smart terminal to ensure that the control commands will be transmitted to the terminal devices. Besides, the home gateway accesses the Internet via Wi-Fi in the station mode as an Access Point, in order to connect to the router and communicate with the web server based on the TCP protocol. The hardware of home gateway is shown in Figure 3.

### 3.3. Web Server

As the core part of whole system, the web server adopts the C/S access mode, and aside from transmitting data, it performs the following functions in the smart home system:

*Transmitting data*: the Web receives the attitude data of mobile phone and the RSSI values of iBeacons, and sends instructions corresponding to the processing results to the home gateway.

*Location computing*: the web server uses the RSSI values of iBeacons to match with the fingerprint database dynamically, thus determining the mobile phone’s location. The related algorithm will be explained in Section 4.

*Pointing computing*: After detecting the mobile phone’s location, the pointed electric device can be determined by applying knowledge of space analytic geometry to analyzing the location data and the rotation data from the magnetometer and inertial sensor. The specific formulas will be given in Section 4.

### 3.4. Android App

*Attitude of mobile phone*: The deflection angle α and pitch angle β are detected by the mobile phone sensor and Earth induction devices.

*Testing communication*: the App can check currently searched information of iBeacons and terminal devices. It can also judge if the communication system is normal via the “hello” signals sent by the web server.

*Setting nodes*: To establish a complete positioning coordinate system, the coordinates of iBeacons and terminal devices must be initialized on the mobile phone’s app.

*Controlling devices*: The electric devices that the user is pointing to are displayed on the control page of the app. When the pointing switch is turned on, the pointing algorithm will be started; otherwise, system will maintain the current pointed devices one per page. Moreover, the page also gives a list of devices that are sorted from near to far by the distance from the phone in the user’s pointing direction. It helps to improve the users’ experience of personalized control, and deals with the situation where several devices are on the axis of the pointing direction at the same time.

### 3.5. IBeacons

**Figure 4:**
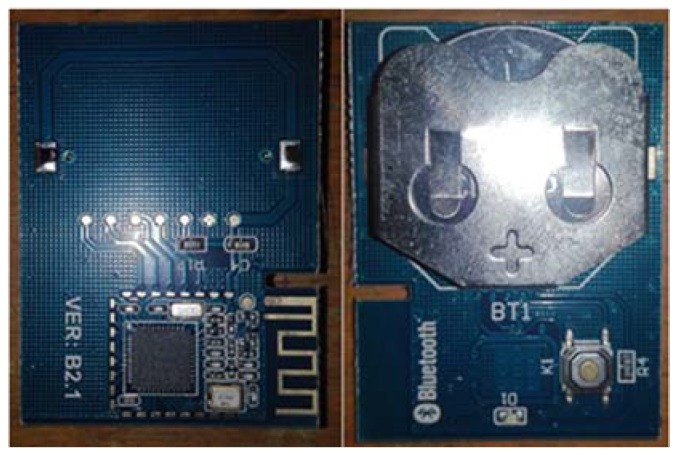
IBeacon.

Briefly speaking, beacon is a signal emission point. IBeacons can broadcast Bluetooth signals carrying their information continuously, and the mobile phone app can detect the signal and transform the signal into RSSI values. The hardware of iBeacon is shown in Figure 4.

## 4. Algorithm Design

### 4.1. Location Computing

The localization algorithm in this model is established on the assumption that the number of iBeacons is certain, which is set to n. Though it is proposed that increasing the number of iBeacons will improve the location accuracy in [17,18], it will decrease the algorithm speed and increase the cost of the system. Therefore, the number of iBeacons in the smart home system is usually held stable at a certain value. This section will propose a more efficient method on the above condition.

Considering the signal intensity of a certain point is random, the Weibull function is applied for describing the Bluetooth signal strength distribution in [19], and the best location is determined via the criterion of maximum probability. Meanwhile, in [20,21,22], it is concluded that the probability distribution of signal strength at a certain point will have various forms and even if the signal strength can be represented by the same probability distribution, the variance is uncertain. However, the Friss transmission equation [12] tells us that RSSI has theoretical expectation at a certain point. Following is the transmission equation:

(1) $$P_{r}\left(d\right)=P_{r}\left(d_{0}\right){\left(\frac{d}{d_{0}}\right)}^{-n}$$

In the formula, $P_{r}$ stands for the power of received signals; Both *d* and $d_{0}$ represent the transmission distance, but the former is the independent variable in the formula, while the latter has been known and established in the experiment. In other words, the path loss corresponding to *d* is the dependent variable, while the path loss corresponding to $d_{0}$ is the actual value. Exponent n is usually between two and five. Taking the logarithm of Equation (1):

(2) $$P_{r}\left(d\right)\left(\mathrm{dB}\right)=P_{r}\left(d_{0}\right)\left(\mathrm{dB}\right)+10n\mathrm{lg}d_{0}-10n\mathrm{lg}d$$

Equation (2) shows the theoretical relationship between the signal strength and the distance. Considering the character of random parameters and sensitivity of wireless channels, in this model Equation (2) is corrected as follows:

(3) $$P_{ri}\left(d\right)\left(\mathrm{dB}\right)=A_{i}\left(\theta ,\varphi \right)-B_{i}\left(\theta ,\varphi \right)\mathrm{lg}d$$

where *i* means the corresponding iBeacons (1≤i≤n). θ,φ are parameters of the spherical coordinate system that takes the corresponding iBeacon as the origin. $A_{i}\left(\theta ,\varphi \right)$ and $B_{i}\left(\theta ,\varphi \right)$ are pending functions. Use coordinate transformation formulas:

(4) $$\{\begin{array}{c} x=d\sin\varphi \cos\theta \\ y=d\sin\varphi \sin\theta \\ z=d\cos\varphi \end{array}$$

Bring Equation (4) into Equation (3) as follows: Formulas (3) and (5) are essentially the same, just going from a polar coordinate to a more convenient rectangular coordinate system:

(5) $$P_{ri}\left(x,y,z\right)=A_{i}\left(x,y,z\right)-B_{i}\left(x,y,z\right)\mathrm{lg}\left(\sqrt{x^{2}+y^{2}+z^{2}}\right)$$

In the actual system, the signal strength of each iBeacon is collected several times in a rectangular parallelepiped space at a spacing of 10 cm, and the average value of each certain point is recorded. The spatial sub database of each iBeacon is the fingerprint matching database of the iBeacon. These values are applied to the least-square learning training on the $A_{i}\left(x,y,z\right)$ and $B_{i}\left(x,y,z\right)$ of Equation (5), in order to find the best fitting $P_{ri}\left(x,y,z\right)$. All the n iBeacons’ fingerprint matching databases form the whole fingerprint matching database, namely the matching matrix $P_{r}\left[n\right]={\left[P_{r1},P_{r2},\ldots ,P_{rn}\right]}^{T}$. Assuming the set of RSSI values that mobile phone received is $R\left[n\right]={\left[R_{1},R_{2},\ldots ,R_{n}\right]}^{T}$, the best location can be determined via the least squares criterion, and following is the criterion:

(6) $$\left(x,y,z\right)\leftarrow \mathrm{argmin}\overset{n}{\underset{i=1}{\sum }}{\left(R_{i}-P_{ri}\right)}^{2}$$

The minimum Euclidean distance from the data measured by mobile phone to the data from database can be calculated based on Equation (6) via iterative fingerprint matching algorithm. The output parameters (*x*, *y*, *z*) stand for the current location of the mobile phone.

### 4.2. PDR Modified Approach

The full name of PDR technology is Pedestrian Dead Reckoning, a trajectory estimation method [23]. Its basic principle is acquiring the initial location of the object firstly, and then detecting the motion trajectory via motion tracking technology. When the PDR is applied to an indoor location, it has two shortcomings: first, the initial location is inaccurate; secondly, the error of motion trajectory is accumulating as distance is increasing. In order to make use of the advantages of the PDR, this model applies the PDR to motion detection, not to location. When the PDR module detects that the object is not moving, the web will call the latch function automatically, and record the RSSI values at that point during a period of time. As is known from the analysis above, the RSSI values at a certain point can be described by probability distribution, and the variance is limited. Therefore, we use law of large numbers [24] as follows:

(7) $$\underset{n\to \infty }{\lim}P\left\{\left|\frac{1}{n}\overset{n}{\underset{k=1}{\sum }}x_{k}-\frac{1}{n}\overset{n}{\underset{k=1}{\sum }}Ex_{k}\right|<\varepsilon \right\}=1,\;\forall \varepsilon >0$$

This helps increase the probability that the measured data will tend to the theoretical mean, thus solving the problems of the time variation of RSSI. Usually, the sample frequency of RSSI values is fixed. If RSSI values are measured as samples, the number of samples will increase with time. Thus, the probability that the mean of samples approaches the mathematical expectation is growing. After adding the PDR correction to the system, the longer the object maintains stationary, the accuracy is higher. The test results will be shown in detail in Section 5.

### 4.3. Gesture Recognition

Gesture recognition is intended to get the inclination angle of the mobile phone, accordingly determine the direction that the user is point to. This module can offer the web server the deflection angle α, pitch angle β and roll angle γ, among which the main angles are β and γ. Supposing the output of magnetic field of the sensor is ($A_{x},A_{y},A_{z}$), its specific calculation formulas are referenced in [9].

## 5. System Test

### 5.1. System Test Environment

**Figure 5:**
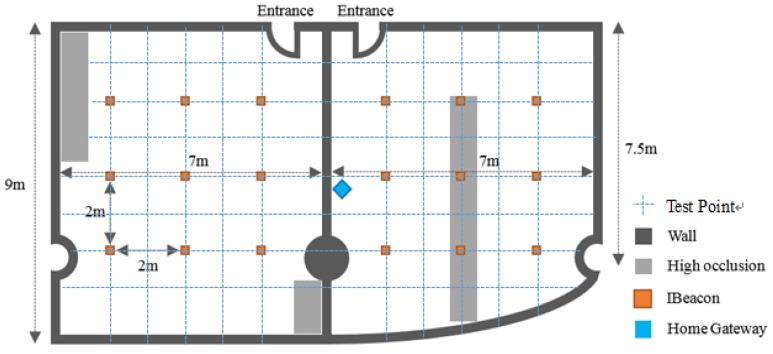
Top View of the Laboratory.

Experiments were conducted at the Laboratory of Science and Technology of Bell Honor School, Nanjing University of Posts and Telecommunications. We selected two rooms with a representative indoor layout for system installation (the process and environment of the installation will be illustrated in detail below). By representativeness we mean that the indoor environment is similar to the general home indoor layout. The top view of the laboratory is shown in Figure 5. The user in the experiment uses a mobile phone with normal an Android system. Terminal devices are installed on the electric devices, such as the access control, lights, and air conditionings. Also, it should be pointed out that this experiment was carried out under the condition that the terminal devices were stationary. Several common use cases encountered during daily life activities were simulated.

**Figure 6:**
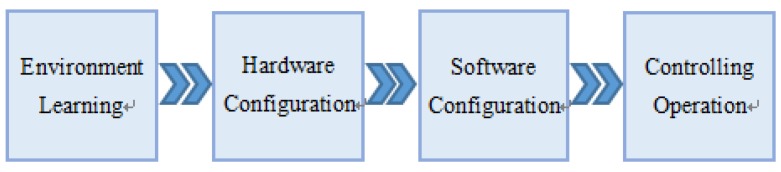
The Process of System Installation and Test.

Figure 6 shows the whole process of the system installation and test. Firstly, environment learning should be conducted. The opened iBeacons are installed on the vertices of the 2 × 2 square array with border length of 2 m on the ceiling of the two rooms. The RSSI values of all the 18 iBeacons form a matrix. The value of each iBeacon is collected 400 times and the average is put into the fingerprint matching database for the web server to use. Secondly, the hardware setup contains two steps: (a) Connect the terminal devices to the electric devices that need to be controlled; (b) Supply power to the home gateway. If the LED on the gateway is blinking, it indicates that the startup is normal. Thirdly, the software setup includes web server startup and mobile device configuration. Mobile device configuration needs to install the app on the mobile phone firstly. Then, we open the app and input the coordinate position of the iBeacons and terminal devices to establish a coordinate system. Geomagnetic correction is then conducted to reduce the error between the geomagnetic coordinate and the iBeacon coordinates.

### 5.2. The Performance Test

The method proposed in this paper focuses on an integrated system, rather than an algorithm or an idea, so the system performance is evaluated from different perspectives, including the system’s stability, accuracy, endurance, and complexity, etc. This section will analyze these typical indicators one by one, and give the corresponding test conclusions to prove the advantages of this system in the field of smart home applications. First of all, the cost and endurance of this system will be explained. The major functional components is sorted in detail as in Table 1.

It should be pointed out that in order to realize the function of each module, the selected device is more than one. The devices used in the experiment are relatively common devices, and the price is the actual purchase price. Also, according to the “barrel principle”, the system’s endurance ability is determined by the device with the shortest normal service life, namely, iBeacons that can work for 1–3 years. In terms of costs, the total price of the system is around 242.5 yuan, which is totally acceptable for common life.

The use complexity represents the complexity of the operations when using the system and is also hard to quantify. If the user needs to set some parameters frequently, or the system startup process is complicated, these will greatly reduce the user experience. In this system, when the positions of electric devices are changed, the user only needs to manually input the modified information to the mobile phone. Except this, all the installation and parameter setup process of the system are one-time settings, that is, the user only needs to complete these settings before the first time use. Therefore, the using complexity of this system is low.

We use experiment data to illustrate the system accuracy. Although there are many indoor location system models for smart home application, they usually have different application scenarios. The results of experiments conducted under different application scenarios cannot be used for direct comparison. Otherwise, it is unfair. Therefore, the accuracy comparison between different systems will be combined with different application scenarios, and will be illustrated in detail in the next part. This part focuses on the binarization least-squares (BLS) matching proposed by [9] whose application scenario is similar to that of this system, classical Friss formula combined with least-squares matching (FLS), PDR and the proposed method in this paper (FLS + PDR), and multi-angle location errors from time to space are compared to illustrate the optimization of the method of this system.

**Figure 7:**
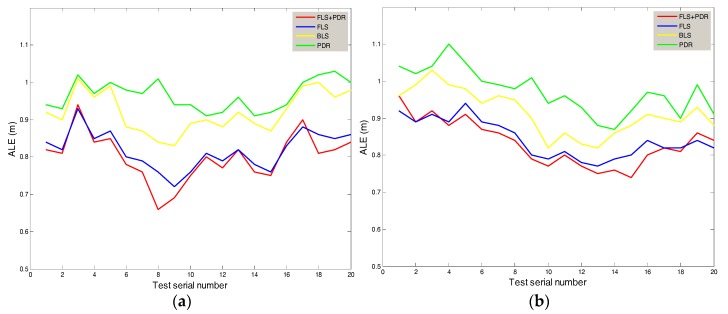
The Relationship between ALE & Test Serial Number (**a**) Area A; (**b**) Area B.

As shown in Figure 5, we set the area of the home on the left side to Area A and the area on the right side to Area B. Figure 7 shows the absolute location errors (ALE) of Area A and Area B, which were measured while the user remained stationary. It can be seen that there are differences in the curve of the same method between A and B, which is due to the heterogeneity of the experiment. The location error of the proposed method in this paper is smallest. The average error of Area A was 0.8 m, and the average error of B was 0.832 m. The method with biggest error is the PDR, which is mainly due to the cumulative effect of additive errors. Compared with BLS and FLS, the method of this system reduced the average error by 11% and 12%.

Table 2 shows the variances of positioning errors (VLE) of Area A and Area B, which were measured while the user remained stationary. The variances are used to measure the stability of positioning at different locations. The variance of errors of the proposed method in this paper was around 0.004, which means that the test data was less volatile than that of the other three methods. In terms of stability, the method of this system reduced the variance of errors by 59.1% and 18.2% compared with PDR and BLS.

**Figure 8:**
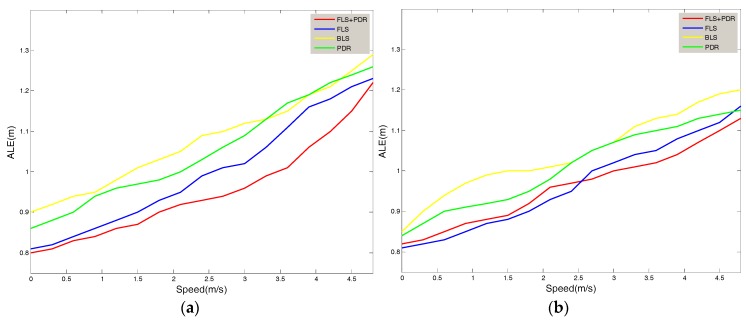
The Relationship between ALE & Speed of Users (**a**) Area A; (**b**) Area B.

Figure 8 shows the relationship between the location errors of the Area A and Area B and the user’s movement speed. It was tested while the user passed a sampling point at a constant speed. It can be seen that the errors of the four methods were monotonically increasing with the user’s speed of movement. The proposed method in this paper still has good location accuracy in the case of high speed movement. At the highest speed of 4.7 m/s, the test error of Area A reached 1.2 m, and the test error of Area B reached 1.13 m. Compared with BLS and FLS, the method of this system reduced the average error by 11% and 2% on the condition of movement.

**Figure 9:**
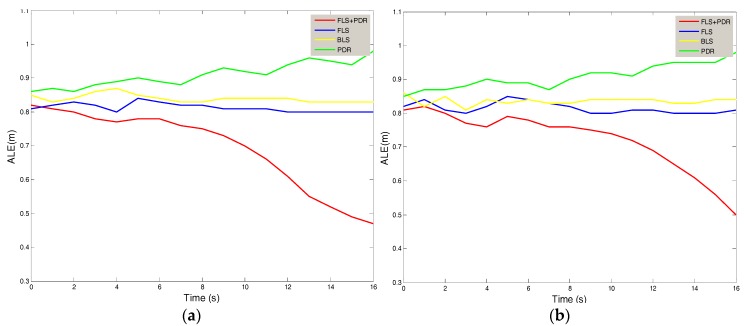
The relationship between ALE & time. (**a**) Area A; (**b**) Area B.

Figure 9 shows the relationship between the absolute location errors (ALE) of the Area A and Area B and the time while the user remained stationary. It can be seen that in the model proposed in this paper, the improved effect of PDR technology on FLS becomes more pronounced over time. If the user keeps stationary for 5 min, the error of this model will decrease by 2%; if the user keeps stationary for 15 min, the error of this model will decrease by 31%.

In addition, the system has good scalability in various application scenarios of smart homes: opening the door remotely, control electric appliances to do housework remotely, or turn off the lighting and air conditioning before going to bed and so on. This series of application scenarios were included in the above system tests, and had excellent performance in various service indicators.

### 5.3. Comprehensive Comparison

**Figure 10:**
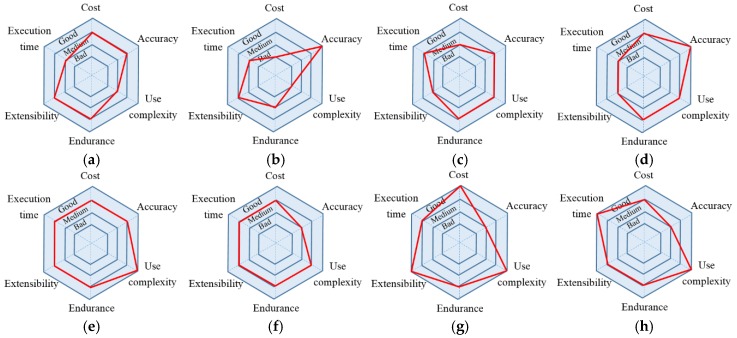
The comparison of all the solutions. (**a**) AR + SD; (**b**) AMR; (**c**) LIFI; (**d**) UWB; (**e**) The proposed method; (**f**) FLS; (**g**) PDR; (**h**) BLS.

Though there are many indoor location solutions, each of them has its own advantages and corresponding application scenarios. Combining these solutions and the location methods compared in the previous part, a group of charts are displayed in Figure 10 to compare all the solutions intuitively. The larger the diameter of the red circle, the better the performance of the system. For example, though the Activity Recognition and Semantic Description (AR + SD) proposed by [25] for indoor location has great accuracy, the system requirements are high and the use operations are complex compared with the solution in this paper. 

Awad [26] put forward a dynamic location solution using autonomous mobile robots (AMRs) for real-time data collection. This solution has a very high accuracy, but the drawbacks are high cost and limited system life. Huang [27] proposed to use LIFI to improve the WIFI indoor location. This method can improve the system performance to some extent but using LIFI increases the cost. Also, in terms of the application scenario, users may need the same smart home control when no light exists, and the solution presented in this paper has solved this problem. Chen [28] used the UWB technology which is now popular for indoor location. The accuracy can be controlled within 0.2 m. However, the disadvantages are low efficiency of instruction execution and limited coverage.

## 6. Conclusions

This paper proposes an improved iBeacon-based location system for smart home control, which is based on the low-energy BLE Beacon technology. IBeacons are combined with the IoT technology to achieve remote equipment control. The proposed system consists of five main parts: web server, home gateway, intelligent terminal, smartphone app and BLE beacons. In the core processor, fingerprint matching based on RSSI stochastic characteristic and posture recognition model based on geomagnetic sensing are used to establish a more efficient equipment control system, and the average indoor location error can be controlled at 0.116 m. Pedestrian Dead Reckoning (PDR) technology is used to improve the location accuracy, and the longer time the target is at rest, the better effect the system has. A personalized menu of remote “one-click” control is finally implemented successfully and the test results are satisfactory to meet the users’ experience. A notable future work task is to study what factors affect the RSSI signal, such as time, temperature and so on in order to build a dynamic, adaptive-learning fingerprint matching library, which can greatly improve the positioning accuracy and the users’ experience.

## Figures and Tables

**Table 1:** Device List.

Device Type	Function	Costs (Yuan)	Battery Life (Year)
CC2541	iBeacon	5 × 18	1~3
STM32F030	Smart Terminal	16 × 4	Sustainable power
YLZ-W2F	Smart Terminal	12 × 4	Sustainable power
YLZ-W2F	Home Gateway	12	Sustainable power
STM32F103	Home Gateway	16	Sustainable power
ESP8266	Home Gateway	12.5	Sustainable power

**Table 2:** VLE of Area A and Area B.

	VLE of Area A	VLE of Area B
FLS	0.0041	0.0040
PDR	0.0096	0.0102
BLS	0.0048	0.0051
PDR+FLS	0.0042	0.0039
